# DNA Methylation Negatively Regulates Gene Expression of Key Cytokines Secreted by BMMCs Recognizing FMDV-VLPs

**DOI:** 10.3390/ijms251910849

**Published:** 2024-10-09

**Authors:** Mingzhu Li, Peng Ning, Ruoman Bai, Zhanyun Tian, Shujia Liu, Limin Li

**Affiliations:** College of Veterinary Medicine, Hebei Agricultural University, Baoding 071000, China; 20202200518@pgs.hebau.edu.cn (M.L.); ningpeng9619@163.com (P.N.); bairuomanike@163.com (R.B.); tian15733536186@163.com (Z.T.); sjliu2023@163.com (S.L.)

**Keywords:** mast cells, DNA methylation, virus-like particles, mannose receptor, cytokine, gene expression

## Abstract

Virus-like particles (VLPs) have been studied and used as vaccines to control foot-and-mouth disease (FMD). Mast cells (MCs) express various pattern recognition receptors that recognize pathogens and secrete numerous cytokines to initiate and modulate immune responses. Our previous study showed that bone marrow-derived mast cells (BMMCs) can recognize foot-and-mouth disease virus-like particles (FMDV-VLPs) to differentially express various cytokines and that histone acetylation can regulate the cytokines secreted during BMMC recognition of FMDV-VLPs. To demonstrate the role of DNA methylation in this response process, BMMCs that recognize FMDV-VLPs were treated with azacytidine (5-AZA), an inhibitor of DNA methylation transferase. We prepared FMDV-VLPs as described previously and cultured the BMMCs. The transcription and expression of key cytokines and transcription factors were determined using real-time quantitative PCR (RT-qPCR) and Western blotting. Results showed that pre-treatment with AZA resulted in the increased transcription and expression of tumor necrosis factor α (TNF-α), interleukin (IL)-6, IL-13, and IL-10, while the changes in IL-13 transcription and IL-6 expression were irrelevant to mannose receptors (MRs). Furthermore, analysis of the transcription factors indicated that both the transcription and expression of nuclear factor-kappa B (NF-κB) increased significantly in the AZA pre-treated group, indicating that DNA methylation may also regulate NF-κB expression to modulate TNF-α, IL-13, and IL-6. However, pre-treatment with AZA did not alter the expression of microphthalmia-associated transcription factor (MITF) or GATA-2. All the data demonstrate that DNA methylation negatively regulates the transcription and expression of TNF-α, IL-13, IL-10, and IL-6 secreted by recognizing FMDV-VLPs. These results provide new ideas for the mast cell-based design of more effective vaccine adjuvants and targeted therapies in the future.

## 1. Introduction

Foot-and-mouth disease (FMD) is an acute, virulent infectious disease caused by the foot-and-mouth disease virus (FMDV), which can infect cattle, pigs, sheep, goats, and other even-hoofed animals, resulting in significant economic losses to the global livestock industry [1,2]. Immunization is an important measure for the prevention and control of FMD [3]. Recently, foot-and-mouth disease virus-like particles (FMDV-VLPs) have exhibited strong potential as candidates for FMD vaccines [4,5,6]. Research in our lab showed that bone marrow-derived mast cells (BMMCs) can recognize FMDV-VLPs via mannose receptors (MRs), and the levels of cytokines, including tumor necrosis factor α (TNF-α), IFN-γ, etc., showed significant differences. In addition, in our previous study, we found that the iMR-VLP group was significantly downregulated (*p* < 0.05), and the level of cytokines, such as interleukin (IL)-9 and IL-10, were further upregulated in the iMR-VLP group [7]. We hypothesized that epigenetic mechanisms may influence the differential expression of cytokines during BMMCs recognizing FMDV-VLPs.

Epigenetics refers to the effects on gene transcription that are independent of changes in DNA sequence. The major and most extensively investigated epigenetic mechanisms include DNA methylation, histone modification, and the effects of non-coding RNAs [8,9]. Increasing evidence has demonstrated that DNA methylation regulates gene expression and plays a vital role in cell differentiation and disease development [10,11,12,13]. DNA methylation at the fifth position of cytosine is catalyzed by DNA methyltransferases (DNMTs), including DNMT1, DNMT3a, and DNMT3b. DNMT1 is involved in maintaining DNA methylation patterns during replication, whereas DNMT3a and DNMT3b are involved in de novo methylation [14]. In general, the DNA methylation of gene promoters downregulates gene expression, whereas DNA demethylation typically upregulates gene transcription [10,15]. The level of DNA methylation can be modified using special drugs such as 5-Azacytidine (5-AZA), a long-term inhibitor of 5-DNA-methyltransferase enzymes [16]. Changes in DNA methylation and the chromatin structure of pro-inflammatory cytokines (IL-1β, IL-6, and TNF-α) are stimulated by LPS in broiler peripheral blood mononuclear cells [17].

Considering their proximity to the external environment, mast cells (MCs), together with macrophages and dendritic cells, constitute the first line of interaction with invading pathogens [18]. MCs are equipped with a wide array of pattern recognition receptors (PRRs) in order to recognize invading pathogens, and can promptly release numerous biologically active mediators when activated, exerting an important role in regulating innate and adaptive immunity [18,19,20]. TNF-α from MCs is required to efficiently initiate dendritic cells in the airway and skin migration to the draining lymph nodes, as well as to recruit T cells in the circulating state [21,22]. CD40/CD40L-mediated MC/B cell contact, together with IL-6 from MCs, directs B cells to differentiate into CD138+ plasma cells and induces IgA production [23]. IL-10 suppresses mast cell-mediated host immune responses and even mediates immunosuppression in acute FMDV infection in swine, as well as persistent FMDV infection in cattle [24,25]. In addition, MCs degranulated and secreted TNF-α, IL-6, membrane cofactor of proteolysis (MCP)-1, and IL-13 in response to bacterial infection [26]. Degranulation products of MCs may act as adjuvants to induce immune responses in mice [27]. A previous study showed that histone acetylation regulates the expression of cytokines in BMMCs by recognizing FMDV-VLPs [28]. The mechanism by which DNA methylation regulates cytokine transcription and expression in BMMCs recognizing FMDV-VLPs remains unknown.

Therefore, 5-AZA, an inhibitor of DNA methylation, was used in this study to demonstrate the regulatory role of DNA methylation in cytokine transcription and expression during bone marrow-derived mast cells recognition of FMDV-VLPs.

## 2. Results

### 2.1. Generation and Characterization of Virus-like Particles

**Figure 1:**
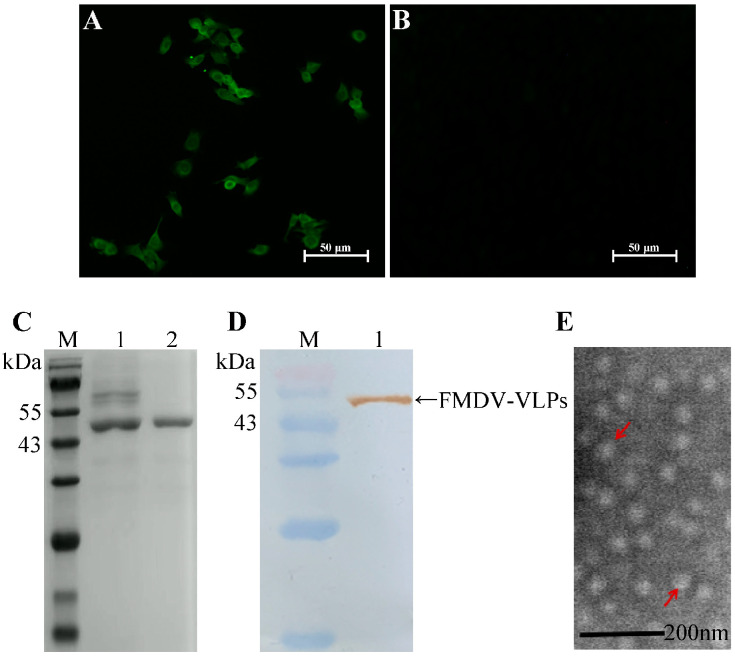
Expression and identification of FMDV-VLPs. (**A**) CHO-K1 cells transfected with recombinant plasmid. (**B**) CHO-K1 cells without being transfected. (**C**) Purification and identification of FMDV-VLPs by SDS-PAGE. M, prestained protein ladder; 1, unpurified supernatants of CHO-K1 cells transfected with recombinant plasmid; 2, purified supernatants of CHO-K1 cells transfected with recombinant plasmid. (**D**) Purification and identification of FMDV-VLPs by Western blot. M, prestained protein ladder; 1, purified supernatants of CHO-K1 cells transfected with recombinant plasmid. (**E**) Identification of FMDV-VLPs by transmission microscopy. Virus-like particles approximately 30 nm in diameter were observed (indicated by red arrows). Full-length blots are presented in Figure S1.

The recombinant plasmid, pCMV-HA-HBcAg-VP1-VP4, was successfully transfected into CHO-K1 cells (Figure 1A,B). At 48 h post-transfection (hpt), the supernatant of the CHO-K1 cells was collected and purified. Purified recombinant proteins were identified using SDS-PAGE (Figure 1C) and Western blotting (Figure 1D). Following this, transmission electron microscopy was employed to characterize the structure of VLPs. As shown in Figure 1E, FMDV-VLPs were nearly spherical and approximately 30 nm in diameter.

### 2.2. Determination of 5-Azacytidine (5-AZA) Dose on BMMCs

**Figure 2:**
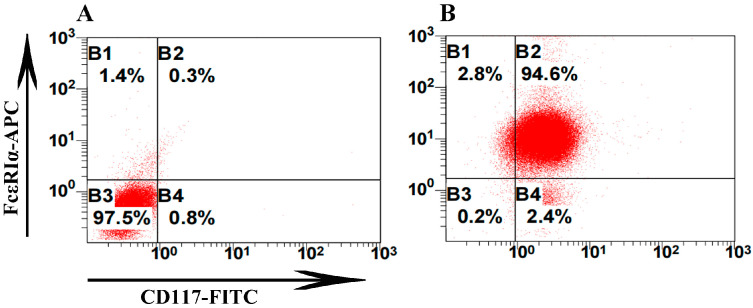
Identification of BMMCs by flow cytometry. BMMCs were identified by flow cytometry at 8 weeks post-culture. (**A**) Allophycocyanin (APC)Armenian Hamster IgG Isotype control antibodies (BioLegend, San Diego, CA, USA) and fluorescein isothiocyanate (FITC) Rat IgG2b, κ isotype control antibodies were used as isotype controls (BioLegend, San Diego, CA, USA). (**B**) BMMCs were directly labeled with APC Armenian Hamster anti-mouse FcεRIα antibodies and FITC Rat anti-mouse CD117 (c-kit) antibodies and identified using flow cytometry. BMMCs, bone marrow-derived mast cells.

Isolated cells from bone marrow were cultured for 7 weeks with the addition of rSCF and rIL-3, and then identified by flow cytometry using FcεRI and CD117 (c-Kit) antibodies. The percentage of c-Kit^+^FcεRI^+^ cells was 94.6%, suggesting that these cells can be used as BMMCs (Figure 2).

**Figure 3:**
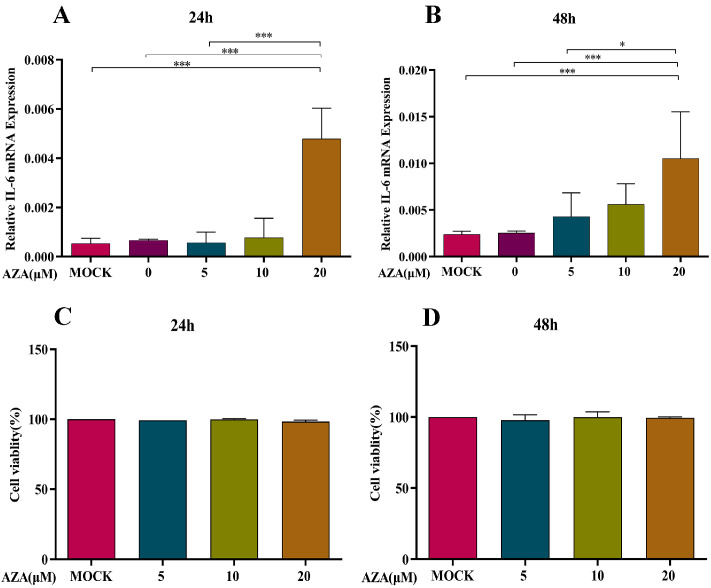
Determination of 5-AZA dose and effect on BMMC viability. The relative expression of IL-6 was detected by real-time quantitative PCR after 5-AZA treatment for 24 h (**A**) and 48 h (**B**), respectively. Subfigures (**C**,**D**) represent the BMMC viability of 5-AZA treatment for 24 h and 48 h, respectively. For cell viability, there were no significances between any two groups. The BMMCs were incubated with FMDV-VLPs for 6 h before 5-AZA treatment. Data are representative of three experiments. * *p* < 0.05, *** *p* < 0.001.

To determine the optimal concentration of 5-AZA, the BMMCs were treated with different concentrations of 5-AZA for 24 h or 48 h, along with the two growth factors mentioned above. The BMMCs were then incubated with VLPs for another 24 h. Real-time quantitative PCR showed that the expression of IL-6 increased in a dose-dependent manner, with an increase in the concentration of the DNA methyltransferase inhibitor when the cells were treated for 48 h. At 20 μM, for both 24 h and 48 h, the expression of IL-6 was significantly different from that in the mock group (*p* < 0.001) (Figure 3A,B). The results of the BMMC viability assay showed that 5-AZA at 0, 5, 10, and 20 μM had no influence on BMMC viability at 24 h or 48 h (Figure 3C,D). Therefore, 20 μM 5-AZA was used in subsequent experiments for 24 h.

### 2.3. DNA Methylation Inhibited the Transcription of Key Cytokines in BMMC Recognition of FMDV-VLPs

**Figure 4:**
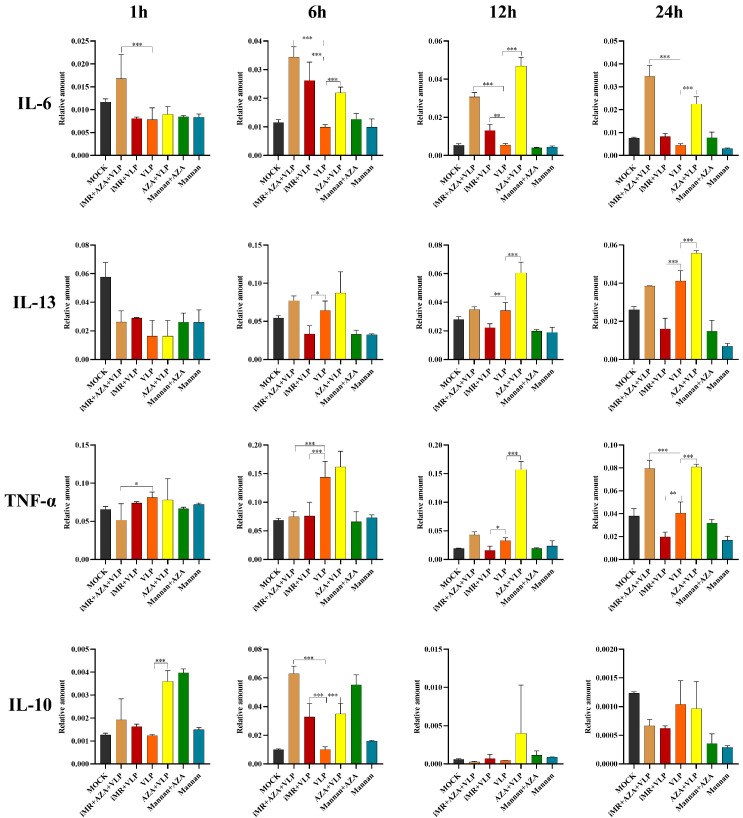
Determination of cytokine transcription by RT-qPCR in different treatment groups. BMMCs were treated with 20 nmol/mL 5-AZA for 24 h. Bone marrow-derived mast cells were harvested for mRNA analysis. The mock group represents pure BMMC culture, while iMR + AZA + VLP represents BMMCs inoculated with mannan, then treated with 5-AZA, and finally added with VLPs. iMR + VLP represents BMMCs inoculated with mannan and then inoculated with VLPs. VLP represents BMMCs inoculated with VLPs. AZA + VLPs represents BMMCs treated with 5-AZA and then inoculated with VLPs. Mannan + AZA represents BMMCs treated with mannan and then inoculated with 5-AZA. Mannan represents BMMCs inoculated with mannan. AZA refers to 5-AZA. Data are presented as Mean ± SD from three independent experiments and normalized to GAPDH. Standard curves of the real-time quantitative PCR are summarized in Figure S2. * *p* < 0.05, ** *p* < 0.01, *** *p* < 0.001.

BMMCs were collected and used to detect the transcription of *IL-6*, *IL-10*, *IL-13*, and *TNF-α* by RT-qPCR. The RT-qPCR results showed that there was no significant difference in the relative expression of *IL-10* between the VLP and mock groups at any time point (Figure 4). Compared to the VLP group, the relative expression levels of *IL-13* and *TNF-α* in the iMR + VLP group were significantly decreased at 6, 12, and 24 h (*p* < 0.05), whereas *IL-10* showed a significant increase only at 6 h (*p* < 0.001) (Figure 4). Compared with the VLP group, the relative expression of *IL-6* in the AZA + VLP group significantly increased at 6, 12, and 24 h, *TNF-α* and *IL-13* were significantly increased at 12 and 24 h (*p* < 0.001), while *IL-10* was significantly increased at 1 and 6 h (*p* < 0.001) (Figure 4). Compared with the VLP group, the relative expression of *IL-6* in the iMR + AZA + VLP group was significantly increased at 1, 6, 12, and 24 h (*p* < 0.001), while *TNF-α* significantly decreased at 1 and 6 h (*p* < 0.05), but significantly increased at 24 h (*p* < 0.001) (Figure 4).

### 2.4. DNA Methylation Inhibited the Expression of Key Cytokines in BMMC Recognition of FMDV-VLPs

**Figure 5:**
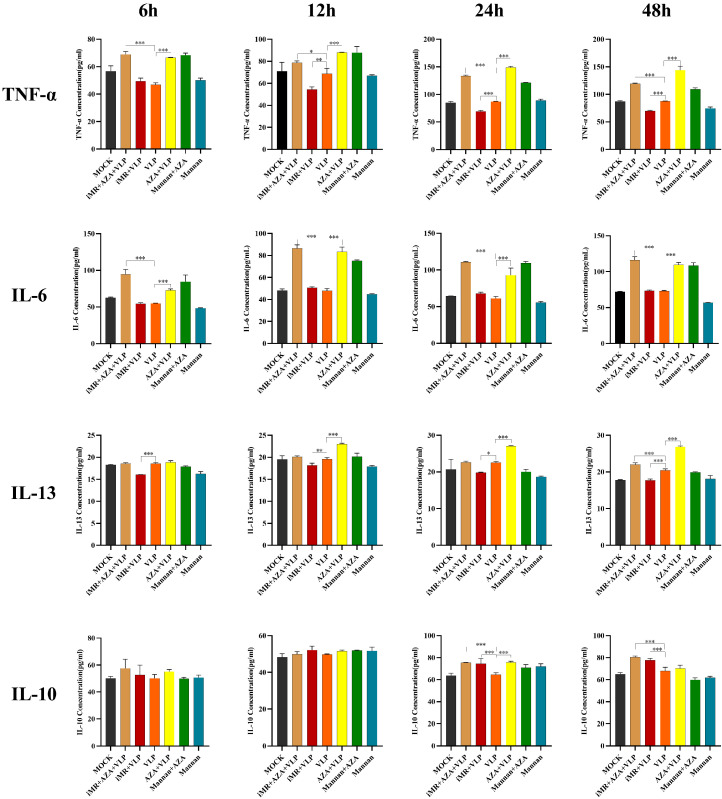
Determination of cytokines expression by ELISA in different treatment groups. BMMCs were treated with 20 nmol/mL 5-AZA for 24 h. The supernatants were collected from the corresponding groups and determined the expression of IL-6, TNF-α, IL-13, and IL-10, respectively. BMMCs without any pre-treatment group are indicated as MOCK. iMR + AZA + VLP represents BMMCs inoculated with mannan, then treated with 5-AZA, finally added with VLPs. iMR + VLP represents BMMCs inoculated with mannan and then inoculated with VLPs. VLP represents BMMCs inoculated with VLPs. AZA + VLPs represents BMMCs treated with 5-AZA and then inoculated with VLPs. Mannan +AZA represents BMMCs treated with mannan and then inoculated with 5-AZA. Mannan represents BMMCs inoculated with mannan. AZA refers to 5-AZA. Data are presented as Mean ± SD from three independent experiments, each with triplicate samples. * *p* < 0.05, ** *p* < 0.01, *** *p* < 0.001.

To determine whether DNA methylation is involved in regulating cytokines expression induced by BMMCs recognizing FMDV-VLPs via MRs, BMMCs inhibited with mannan were pre-treated with 5-AZA, and cytokine ELISA kits were used to examine the levels of IL-6, IL-10, TNF-α, and IL-13. The expression levels of IL-6, IL-13, and TNF-α in cells of the AZA + VLP group were significantly increased compared with those observed in cells treated with VLP alone (*p* < 0.01) at 12 h, 24 h, and 48 h, respectively (Figure 5). Similar results were observed only at 24 h for IL-10. Furthermore, when BMMCs were pre-treated with mannan and 5-AZA (iMR + AZA + VLP), the expression levels of IL-6 and TNF-α were dramatically increased compared to those in the VLP group at all time points (*p* < 0.01) (Figure 5). Similar results were observed for IL-10 and IL-13 at 24 h and/or 48 h (Figure 5).

### 2.5. DNA Methylation Mainly Downregulates NF-κB Expression in BMMC Recognition of FMDV-VLPs

**Figure 6:**
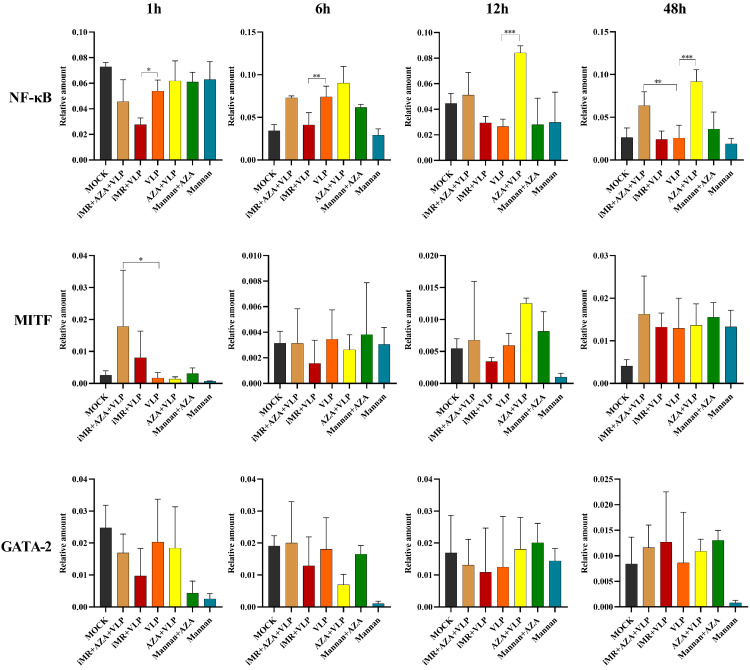
Determination of transcription factors mRNA expression. RT-qPCR was utilized to determine the expression of *NF-κB*, *MITF*, and *GATA-2* in different BMMC groups at different times. BMMCs without any pre-treatment group are indicated as MOCK. iMR + AZA + VLP represents BMMCs inoculated with mannan, then treated with 5-AZA, and finally added with VLPs. iMR + VLP represents BMMCs inoculated with mannan and then inoculated with VLPs. VLP represents BMMCs inoculated with VLPs. AZA + VLPs represents BMMCs treated with 5-AZA and then inoculated with VLPs. Mannan + AZA represents BMMCs treated with mannan and then inoculated with 5-AZA. Mannan represents BMMCs inoculated with mannan. AZA refers to 5-AZA. Data are presented as Mean ± SD from three independent experiments and normalized to *GAPDH*. * *p* < 0.05, ** *p* < 0.01, *** *p* < 0.001.

Transcription factors can interact with nucleosomal DNA to regulate the activation of latent enhancers of immune response genes and initiate gene expression. To determine the role of DNA methylation in the activation of transcription factors in BMMCs recognizing FMDV-VLPs, the transcription and expression of MRs, *MITF*, *NF-κB*, and *GATA-2* were analyzed by RT-qPCR and Western blot. As shown in Figure 6, the transcription of *NF-κB* in the AZA + VLP group was remarkably increased compared with those observed in the VLP group (*p* < 0.01) at 12 h and 24 h, respectively. Only significant increases were observed in the iMR + AZA + VLP group compared to the VLP group at 24 h. The transcription of *MITF* and *GATA-2* showed almost no significant differences between any two groups (Figure 6).

**Figure 7:**
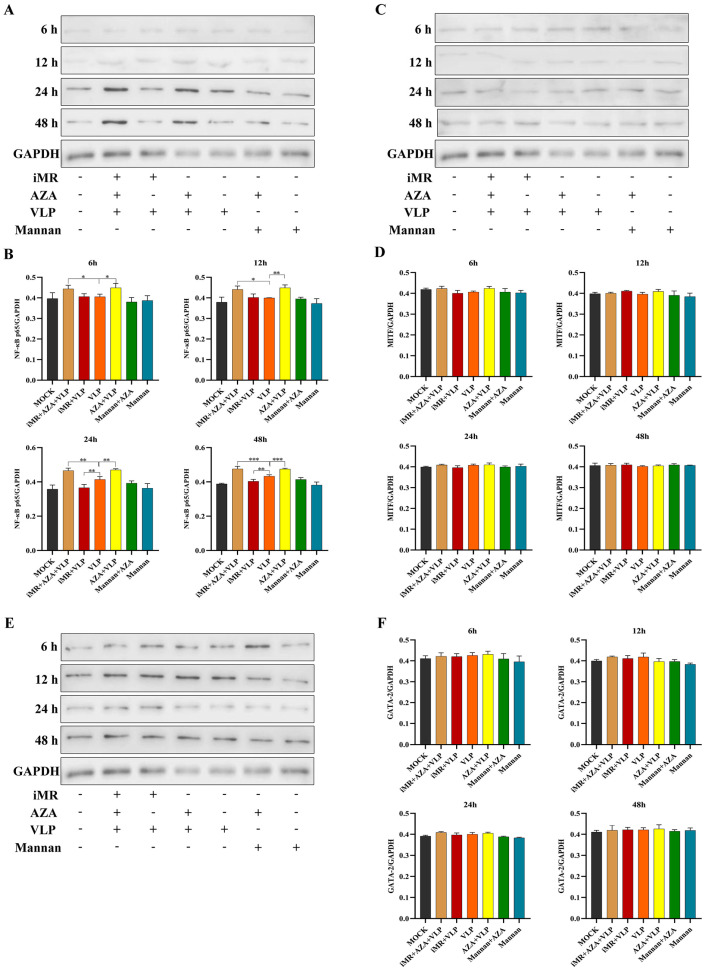
Determination of transcription factors mRNA expression by Western blot. BMMCs without any pre-treatment group are indicated as MOCK. iMR + AZA + VLP represents BMMCs inoculated with mannan, then treated with 5-AZA, and finally added with VLPs. iMR + VLP represents BMMCs inoculated with mannan and then inoculated with VLPs. VLP represents BMMCs inoculated with VLPs. AZA + VLPs represents BMMCs treated with 5-AZA and then inoculated VLPs. Mannan + AZA represents BMMCs treated with mannan and then inoculated with 5-AZA. Mannan represents BMMCs inoculated with mannan. AZA refers to 5-AZA. Data are presented as Mean ± SD from three independent experiments. (**A**,**B**) represents the Western blot results and quantitative analysis of NF-κB p65, respectively; (**C**,**D**) represents the Western blot results and quantitative analysis of MITF, respectively; (**E**,**F**), represents the Western blot results and quantitative analysis of GATA-2, respectively. Full-length blots are presented in Figure S1. * *p* < 0.05, ** *p* < 0.01, *** *p* < 0.001.

The analysis of the Western blot showed that the expression of NF-κB in the iMR + VLP group was significantly decreased compared to that in the VLP group *(p* < 0.01) at 24 h and 48 h. When the BMMCs were pre-treated with AZA, the expression of NF-κB in the AZA + VLP group and the iMR + AZA + VLP group was significantly increased compared to the VLP group at all time points, respectively (*p* < 0.01) (Figure 7). The expression levels of MITF and GATA-2 were not significantly different among the groups (*p* > 0.05). 

### 2.6. Analysis of DNA Methylation Levels and Patterns

**Figure 8:**
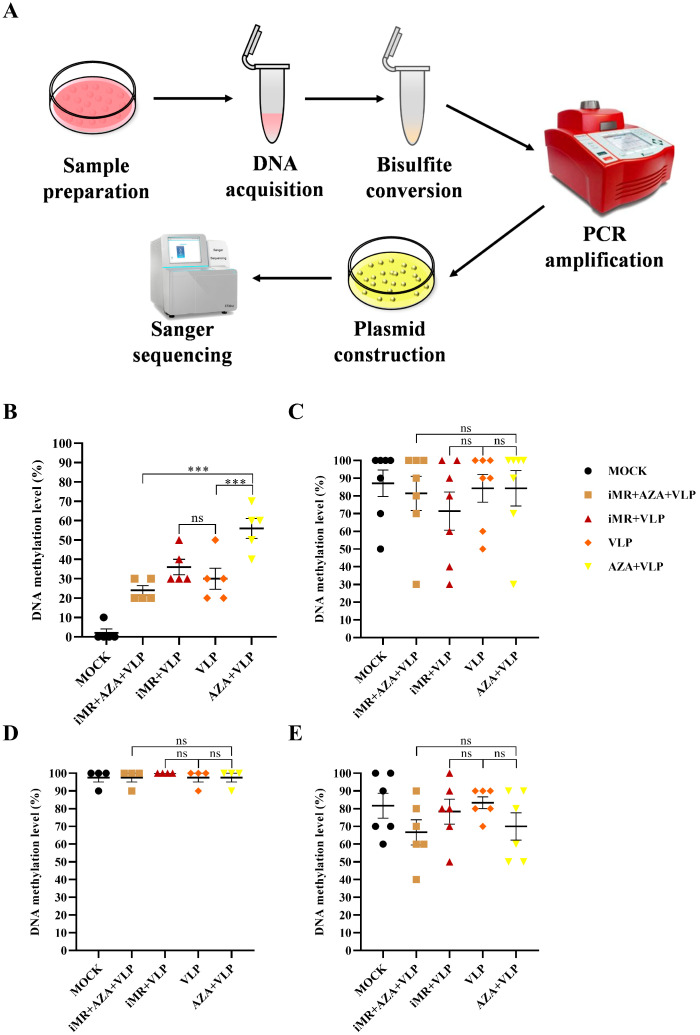
Flow chart of methylation sequencing and statistical analysis of methylation levels in cytokine promoter regions between groups. (**A**) The flow chart of methylation sequencing. (**B**–**E**) represents methylation levels in the *IL-6*, *IL-13*, *TNF-α*, and *IL-10* promotor regions, respectively. BMMCs without any pre-treatment group are indicated as MOCK. iMR + AZA + VLP represents BMMCs inoculated with mannan, then treated with 5-AZA, and finally added with VLPs. iMR + VLP represents BMMCs inoculated with mannan and then inoculated with VLPs. VLP represents BMMCs inoculated with VLPs. AZA + VLPs represents BMMCs treated with 5-AZA and then inoculated VLPs. AZA refers to 5-AZA. Each datapoint represents the average of DNA methylation rate in each locus. DNA methylation rate in each locus = number of methylations at that locus/number of sequences measured. A one-way ANOVA was carried out. *** *p* < 0.001, ns indicates no significance.

**Figure 9:**
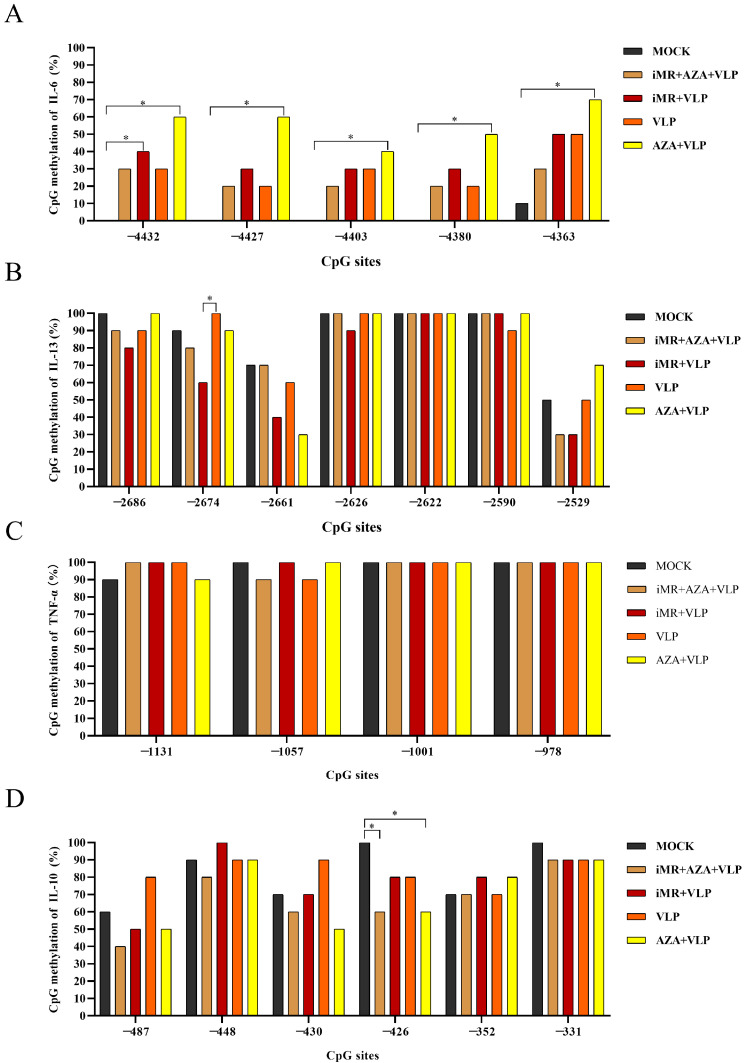
Methylation rate of each locus in the *IL-6*, *IL-13*, *TNF-α*, and *IL-10* promotor regions. (**A**) *IL-6*. (**B**) *IL-13*. (**C**) *TNF-α*. (**D**) *IL-10*. BMMCs without any pre-treatment group are indicated as MOCK. iMR + AZA + VLP represents BMMCs inoculated with mannan, then treated with 5-AZA, and finally added with VLPs. iMR + VLP represents BMMCs inoculated with mannan and then inoculated with VLPs. VLP represents BMMCs inoculated with VLPs. AZA + VLPs represents BMMCs treated with 5-AZA and then inoculated VLPs. AZA refers to 5-AZA. The abscissa number indicates the location of each CpG locus relative to the 5′-UTR. DNA methylation rate in each locus = number of methylations at that locus/number of sequences measured. Z-test was carried out. * *p* < 0.05.

The DNA methylation level and pattern were further explored by determining the CpG island at the gene promotor proximity region (Figure 8A). The DNA methylation pattern is shown in Figure S3. For the *IL-6* promotor, the DNA methylation levels of the iMR + AZA + VLP group and the VLP group were significantly lower than the AZA + VLP group, respectively (Figure 8B). Mannose receptors may be involved in regulating the methylation of IL-6 detection regions −4432, −4427, and −4380 in BMMCs to affect *IL-6* transcription (Figure 9A). There were no statistically significant differences in promotor region methylation levels of *IL-10*, *TNF-a* and *IL-13* between the groups (Figure 8C–E). The methylation rate of locus −2686, −2674, −2661, −2666, and −2529 in the iMR + VLP group decreased compared with that in the VLPs group, especially at locus −2674 (*p* < 0.05) (Figure 9B), suggesting that mannose receptors are involved in DNA methylation at this site and regulate *IL-13* transcription. For *IL-10*, the DNA methylation at locus −487, −430, and −426 in the iMR + AZA + VLP, iMR + VLP, and AZA + VLP groups were lower than those in the VLP group, respectively (Figure 9D). The DNA methylation rate of the promoter region of *NF-κB* was more than 90% in each group, and there was no significant difference. The −1939 locus may be involved in *NF-κB* transcriptional regulation (Figure S4).

## 3. Discussion

In this study, we demonstrated that DNA methylation could regulate the transcription and expression of genes identified in BMMCs that recognize FMDV-VLPs. Mast cells play a vital role in regulating immune responses by secreting various cytokines after stimulation with antigenic agents. Our data highlighted that the role of DNA methylation in gene expression should be considered when designing novel FMDV-VLP vaccines based on mast cells.

DNA methylation is a well-maintained epigenetic process that occurs in mammalian cells. Studies have shown that the unique DNA methylation status of different CD4 + T cells can be used to evaluate memory T cell differentiation [29]. Moreover, DNA methylation exerts a dual effect on the regulation of gene expression. DNA methylation can inhibit transcription by blocking the binding of certain transcription factors to the corresponding promoter, whereas in some situations, TFs can bind methylated DNA to activate transcription [9,30]. Our study showed that the transcription and expression of IL-6, TNF-α, IL-13, and IL-10 were increased in the AZA + VLP group compared to the VLP group, indicating that DNA methylation inhibits the expression of IL-6, TNF-α, IL-13, and IL-10 (Figure 4 and Figure 5). Notably, the effect of IL-10 was only observed at 24 h. It is reported that DNA demethylase inhibition can upregulate the expression of IL-6, TNF-α, and IL-13 in the IgE-activated mouse mast cells [10]. Surprisingly, the methylation levels of the promoter regions of cytokines (TNF-α, IL-13, and IL-10) showed no significant differences among the different treatment groups (Figure 8C–E), despite the AZA + VLP group showing a trend of lower methylation levels than the VLP group at specific sites (Figure 9). The reason for the inconsistency between the overall methylation levels and the gene transcription levels may be partly related to the insufficient number of sequences we measured, and also because the key factor that may affect gene transcription is CpG single-site methylation in the promoter region. In addition, as most DNA methylation sites in the genome are not located in the promotor region [31], treatment with 5-AZA may cause the inconsistency mentioned above.

Mast cells and sentinel cells utilize MRs to recognize pathogens or danger signals to produce a variety of cytokines. Owing to their special location and rapid response to pathogens, mast cells have drawn increasing attention for novel vaccine design [32,33]. When the MR was inhibited, the expression of IL-6, TNF-α, and IL-13 decreased and increased for IL-10 compared to the VLP group. This means that BMMCs recognize FMDV-VLPs to promote the expression of IL-6, TNF-α, and IL-13, but inhibit the expression of IL-10 via MRs (Figure 5). These results indicate that the MR signal pathway plays an important role in the regulation of innate immunity and adaptive immunity, especially maintaining IL-10 at a proper level. Targeting mannose receptors on mast cells may be taken into consideration in FMD novel vaccine design. It is particularly noteworthy that the expression of IL-6 and TNF-α in the iMR + AZA + VLP group at all time points, and IL-10 and IL-13 in the iMR + AZA + VLP group at 24 h and/or 48 h increased significantly compared to the VLP group. Nevertheless, they showed no significant changes compared to the AZA + VLP group (Figure 5). These data suggest that the role of DNA methylation in the regulation of gene expression is more important than that of MRs. It is worth noting that after blocking the mannose receptors on BMMCs and then incubating with VLPs, the methylation levels of IL-10 (−487, −430, and −426) and IL-13 (−2686, −2674, −2661, −2666, and −2529) were downregulated, indicating that the mannose receptor is involved in the regulation of DNA methylation in the promoter region. For the difference in transcription levels between IL-10 and IL-13, the effect of histone acetylation cannot be ruled out [28].

In addition, we demonstrated that DNA methylation inhibited the expression of NF-κB during the process of BMMC recognition by FMDV-VLPs, whereas DNA methylation hardly influenced the expression of MITF and GATA-2 (Figure 6 and Figure 7). NF-κB, a nuclear transcription factor widely found in eukaryotic cells, can bind to open chromatin to initiate a number of cytokines, especially pro-inflammatory cytokines such as IL-6 and TNF-α [34,35]. Our previous ATAC-seq analysis showed that MRs expressed on BMMCs can affect the NF-κB pathway by changing chromatin accessibility to regulate the transcription of specific cytokines, ultimately leading to the differential expression of cytokines [36]. However, the detailed MR signaling pathway of recognizing FMDV-VLPs, including the precise molecular pathways through which MRs influence methylation, remain unclear.

DNA methylation and histone modification are highly intertwined and rely on each other to maintain the epigenetic state of mammalian cells. In our previous study, we found that histone acetylation can downregulate the expression of IL-6, IL-13, and TNF-α during BMMC recognition of FMDV-VLPs [28]. DNA methylation exerts the opposite effect on these cytokines. DNA methylation may be taken as a mechanism for setting up local histone deacetylation and inhibiting chromatin opening, and several transcription factors (SP1, NRF1, AP1, GATA3, MITF, NF-κB, etc.) may bind to the motifs with altered chromatin accessibility to regulate gene transcription when BMMCs recognize FMDV-VLPs [36]; therefore, the detailed mechanism and pathway through which BMMCs recognize FMDV-VLPs via mannose receptors need to be further explored.

## 4. Materials and Methods

### 4.1. Plasmids, Cells, and Mice

The recombinant plasmid, pCMV-HA-HBcAg-VP1-VP4, was constructed and stored in the lab [7,28]. Chinese hamster ovary (CHO)-K1 cells used to prepare the VLPs were obtained from ProCell (Wuhan, China). Eight-week-old specific pathogen-free (SPF) C57BL/6N mice (Certificate No.110011210111475962) were purchased from Beijing Vital River Laboratory Animal Technology Co., Ltd. (Beijing, China). All mice were kept in an independent ventilation system and the breeding conditions were pathogen-free. The mice had free access to water and complete food (Beijing Vital River Laboratory Animal Technology Co., Ltd.). Sterilized shaved bedding was added to each cage. After acclimating for a week, approximately ten mice (sufficient cells were obtained) were euthanized by cervical dislocation and subjected to dissection. Mice were used in accordance with the Laboratory Animal Guidelines for the Ethical Review of Animal Welfare in China (GB/T35892-2018) and the Animal Welfare and Ethics Committee of the Laboratory Animal Center of Hebei Agricultural University (approval code: 2019006).

### 4.2. Preparation of FMDV-VLPs

Transfection and FMDV-VLP purification and characterization were performed as previously described [28]. CHO-K1 cells at a density of 90% were washed with Opti-MEM (Gibco, Carlsbad, CA, USA). The recombinant plasmid, pCMV-HA-HBcAg-VP1-VP4, was transfected into CHO-K1 cells using the liposome transfection method. The ratio of plasmid to lipofectamine 2000 (Invitrogen, Carlsbad, CA, USA) was 1:1 (*w*/*v*, μg/μL). At 24 h post-transfection (hpt), the CHO-K1 cells were fixed and the expression of recombinant plasmid was analyzed by indirect immunofluorescence assay (IFA). At 48 hpt, BMMC culture supernatants were collected and purified. For Western blot analysis, a rabbit anti-recombinant FMDV VP1–VP4 protein polyclonal antibody was used as the primary antibody at a dilution of 1:1000. Subsequently, horseradish peroxidase (HRP)-labeled goat anti-rabbit IgG (ZSGB Bio, Beijing, China) was used as the secondary antibody at a dilution of 1:40,000. For the morphological observation of VLPs, the purified recombinant protein was prepared, negatively stained with 2% phosphotungstic acid, and observed using a transmission electron microscope (TEM).

### 4.3. Preparation and Identification of BMMCs

Murine BMMCs were obtained as described by the previous study [28]. Briefly, cells were isolated from the femurs and cultured in RPMI-1640 medium (Gibco) supplemented with 10% fetal bovine serum and 100 U/mL penicillin/100 μg/mLstreptomycin (Gibco) at 37 °C under 5% CO_2_. Additionally, 10 ng/mL IL-3 (rIL-3, ABclonal, Woburn, MA, USA) and 20 ng/mL stem cell factor (rSCF, ABclonal, Woburn, MA, USA) were added to the complete culture medium. After 7 days, adherent cells were carefully removed from the culture. Non-adherent cells were recovered, centrifuged, and resuspended with fresh culture medium to enhance the purity of the BMMCs. This step was repeated every 7 days until the adherent cells disappeared. After culturing for 7 weeks, the BMMCs were assessed by an analysis of the expression of c-kit (CD117) (BioLegend, San Diego, CA, USA) and FcεRIα (BioLegend, California, USA) on the BMMCs. The BMMCs were used in further studies if their purity exceeded 92%.

### 4.4. Determination of 5-Azacytidine (5-AZA) Dose

In order to determine the working concentration of 5-AZA (Sigma, St. Louis, MO, USA) on BMMCs, the identified BMMCs were suspended in RPMI 1640 with a concentration of 1 × 10^6^ cells/mL and inoculated to a 48-well cell culture plate, with 300 μL per well. The 5-AZA was added to the wells at final concentrations of 0, 5, 10, and 20 μM. The BMMCs not treated with 5-AZA served as blank controls. After incubation for 24 h and 48 h at 37 °C under 5% CO_2_, 20 μg/mL VLPs (final concentration) were added to these wells and incubated for 6 h. The BMMCs were collected and total RNA was extracted. The relative expression levels of IL-6 were determined in different groups.

A commercially available cell viability assay, Cell Counting Kit-8 (CCK-8) (Biyuntian, Shanghai, China), was used to evaluate the cytotoxic effect of 5-AZA in vitro. The BMMCs (1 × 10^6^ cells/mL) were seeded into 96-well plates and incubated with 5-AZA at final concentrations of 0, 5, 10, and 20 nmol/mL for 24 and 48 h at 37 °C in a 5% CO_2_ incubator. Cells not treated with 5-AZA were used as blank controls. All experiments were performed in triplicate. Afterwards, 10 µL of CCK-8 solution was added to each well, incubated for 4 h, and then absorbance was determined at 450 nm using an ELISA reader (Thermo Fisher Scientific, Waltham, MA, USA). The percentage cell viability was calculated using the following equation: cell viability (%) = (mean absorbency in test wells)/(mean absorbency in control wells) × 100.

### 4.5. Real-Time Quantitative PCR

BMMCs (1 × 10^6^ cells/mL) were seeded into 24-well cell culture plates at a density of 5 × 10^5^ cells/well. After adding mannan at a final concentration of 3 mg/mL for 2 h, the cells were treated with 5-AZA (20 nmol/mL) for 24 h, with no mannan as the negative control and ligands with MR as the positive control. Except in the positive control group, 20 μg/mL FMDV-VLPs (final concentration) were added to the wells. BMMCs were collected 1, 6, 12, and 24 h after VLP treatment. Detailed grouping of cell processing was as follows: the mock group (pure BMMC culture), the iMR + AZA + VLP group (BMMCs were inoculated with mannan, then treated with 5-AZA, and finally VLPs were added to the BMMCs), the iMR + VLP group (BMMCs were inoculated with mannan and then inoculated with VLPs), the VLP group (BMMCs were inoculated with VLPs), the AZA + VLPs group (BMMCs were treated with 5-AZA and then inoculated with VLPs), the Mannan + AZA group (BMMCs were treated with mannan and then inoculated 5-AZA), and the Mannan group (BMMCs were inoculated with mannan). Total RNA was extracted using the TransZoL Up (TransGen Biotech, Beijing, China) and a reverse transcription of RNA into cDNA using MMLV (Promega, Madison, WI, USA) from BMMCs and RT-qPCR was performed according to our previous study and the manufacturer’s protocols [28]. Primer’s information is listed in Table 1.

### 4.6. Enzyme-Linked Immunosorbent Assay (ELISA)

BMMC culture supernatants were harvested as described above in the Section 4.4. The content of IL-6, IL-13, TNF-α, and IL-10 in bone marrow-derived mast cell culture supernatants was determined using ELISA kits (R&D Systems, Minneapolis, MN, USA) according to the manufacturer’s instructions.

### 4.7. Western Blot

BMMCs were treated and harvested as described in the Section 2.4. The cells were lysed using a high-efficiency RIPA cell lysate, and the total protein content of the BMMCs was separated using sodium dodecyl sulfate-polyacrylamide gel electrophoresis (SDS-PAGE) and then transferred to a polyvinylidene fluoride (PVDF) membrane. The membranes were blocked with 5% skim milk for 30 min. After blocking, rabbit anti-human MITF antibody (1:500, Huaan, Hangzhou, China), rabbit anti-human NF-κB p65 antibody (1:500, Huaan, Hangzhou, China), rabbit anti-human GATA-2 mAb (1:500, Aibotech, Wuhan, China), and rabbit anti-human GAPDH (1:1000, GeneTex, Irvine, CA, USA) were added to the respective membranes. After incubation at 4 °C for 16 h, the membranes were washed with TBST and incubated with HRP-labeled goat anti-rabbit antibody at a dilution of 1:10,000 for 1 h at room temperature. After washing the membrane with TBST five times, the PVDF membranes were developed using a chemiluminescent color-developing solution and imaged.

### 4.8. Measurement of DNA Methylation Levels in Promoter DNA

Total DNA was extracted from different BMMC samples following the instructions of the Quick-DNA™ Miniprep Plus Kit (Zymo Research, Irvine, CA, USA). A total of 20 μL of each BMMC sample’s total DNA (200 ng) were used for the bisulfite conversion process using an EZ DNA Methylation-Gold kit (Zymo Research, Irvine, CA, USA). According to the promoter sequences of IL-6, IL-10, IL-13, TNF-α, and NF-κB (p65), primers were designed using Methprimer (https://www.urogene.org/methprimer/, accessed on 1 March 2023, Table 2). The bisulfite-treated DNA was taken as a template and a PCR was carried out using ESmix (CWbio, Beijing, China). All PCR products were ligated into a pMD-18T vector (Takara, Beijing, China) and then sequenced. The 10 valid sequencing results of each group were used for methylation analysis.

### 4.9. Statistical Analysis

All data acquired in this study are expressed as means ± SD. A one-way ANOVA, followed by Bonferroni’s Multiple Comparison Test, was performed using IBM SPSS Statistics 26 software, and graphs were generated using GraphPad Prism 9. *p* < 0.05 represents a statistically significant difference, while *p* < 0.01 represents an extremely significant difference.

## 5. Conclusions

This study demonstrates that DNA methylation negatively regulates the transcription and expression of TNF-α, IL-13, IL-10, and IL-6 secreted by BMMCs that recognize FMDV-VLPs. These results provide new ideas for the mast cell-based design of more effective vaccine adjuvants and targeted therapies in the future.

## Figures and Tables

**Table 1:** Primers used in RT-qPCR.

Name	Sequence (5′–3′)	Tm (°C)	Product Size (bp)	Efficiency	R^2^
*GATA-2*	Forward: GGGTGGAACATACTCTTGGC	51	120	1.92	1.00
	Reverse: GCACAGTAATGGCGGAACAA				
*IL-6*	Forward: ACGGAATGGCTAAGGACCAAGAC	53	132	1.97	1.00
	Reverse: GCGGAATGCCCACAAACTGATA				
*IL-10*	Forward: CGCCCTATTTAGAAAGAAGCCCA	52	140	1.99	1.00
	Reverse: AAAGGAAGAACCCCTCCCATCAT				
*IL-13*	Forward: CAAGCAACCAGCCCTCA	50	145	1.91	1.00
	Reverse: TACAACCTCCCCCCATTCA				
*MITF*	Forward: CATCACCTTTACCAACAACCTCG	51	230	1.97	1.00
	Reverse: AACAGGCTCGCTAACACGCA				
*TNF-α*	Forward: AAAGGGAGAGTGGTCAGGTTGC	51	95	2.01	1.00
	Reverse: CTCAGGGAAGAATCTGGAAAGGT				
*NF-κB*	Forward: CATCCTCGTCCGCCTATTAC Reverse: GACTCTCCTCCCTTTCCTTGT	50	93	1.90	1.00
*GAPDH*	Forward: CCTTCCGTGTTCCTAC	45	152	1.73	1.00
	Reverse: GACAACCTGGTCCTCA				

**Table 2:** Primers used in DNA methylation sequencing analysis.

Name	Sequence (5′–3′)	Tm (°C)	Product Size (bp)
*TNF-α*	Forward: ATTTTGTTTGTTTGTGTTTGTTTTG	48	206
	Reverse: CCCTAACTCATCCTTTAAATCTCCT		
*IL-13*	Forward: AGAGTTATTTTGTTGGGGTTTTTTT	53	170
	Reverse: ATTCCTACCTACCCCTCTACTCACT		
*IL-6*	Forward: AATGGTGAAGTATAAGTAGTAGT	46	344
	Reverse: ACCCTATAATTTCATCTCTTCTT		
*IL-10*	Forward: AGAAAGTTAGTTTAAGGGATTTTTT	48	168
	Reverse: CAATCTCCCTACCTAAAAACCTACTAC		
*NF-κB*	Forward1: GTTGTGGGTAGTTGTGGTTA	49	356
	Reverse1: ACAACTTACCAAACTCATTTCTT		
*NF-κB*	Forward2: TTTTTGTTTGATAGGGTGTGAAATA	47	249
	Reverse2: ATCCTAAAAACAAATCAAAATCATC		

## Data Availability

All the data produced for this work are contained within the article.

## Supplementary Materials

The following supporting information can be downloaded at https://www.mdpi.com/article/10.3390/ijms251910849/s1.
