# Supplementary material for: DNA Methylation Negatively Regulates Gene Expression of Key Cytokines Secreted by BMMCs Recognizing FMDV-VLPs
Source: Int J Mol Sci. 2024 Oct 9;25(19):10849. doi: 10.3390/ijms251910849 (PMC11477203; doi:10.3390/ijms251910849)
Supplement: Supplementary file 1 [file ijms-25-10849-s001.zip › Figure S1.pdf]

Supplementary Figure-S1: Uncropped blot images displayed in the context.

Western blot original figure corresponds to the figure annotation in the manuscript.

Western blot: Untreated

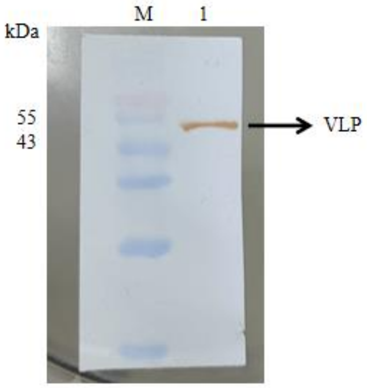

Figure S1A Expression and identification of FMDV-VLPs. M, protein molecular marker; 1, cell supernatants after purification (purified VLPs).

MITF、GAPDH 6 h :

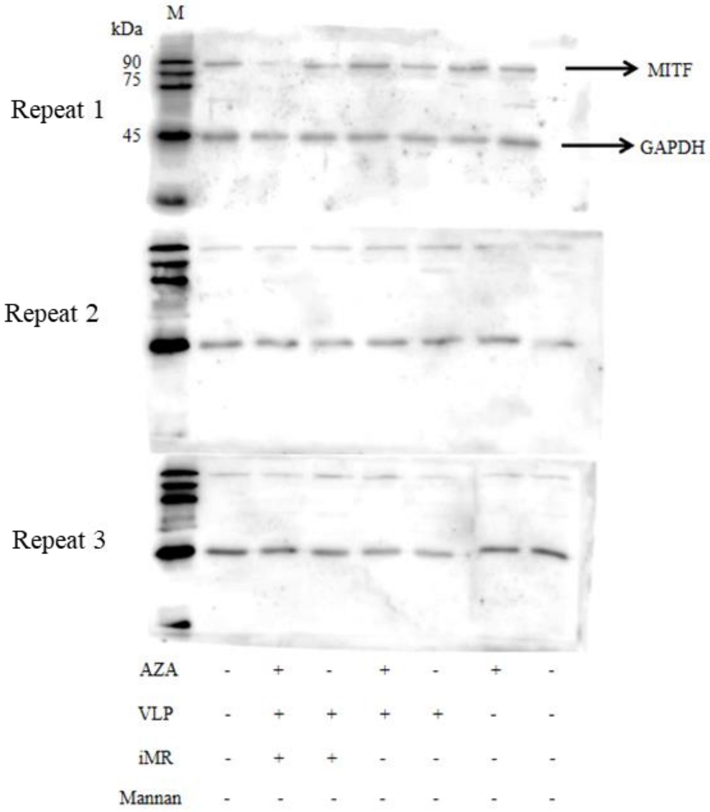

MITF、GAPDH 12 h :

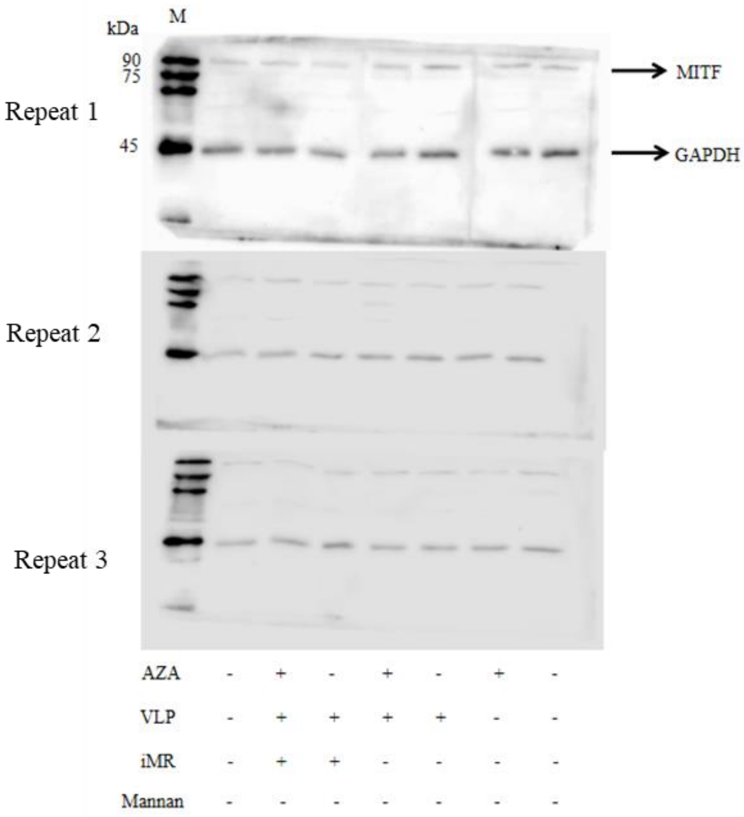

MITF、GAPDH 24 h :

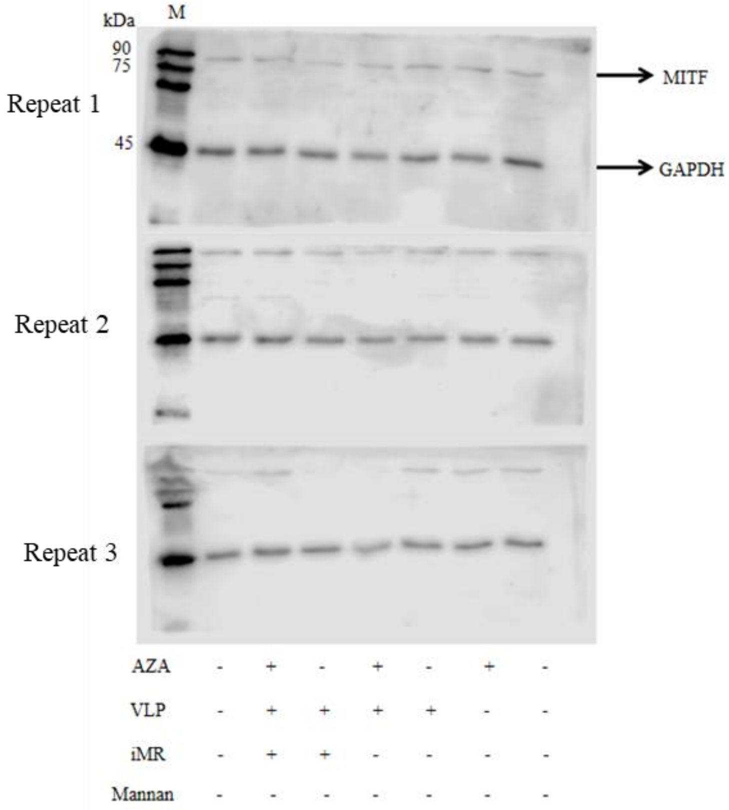

MITF、GAPDH 48 h :

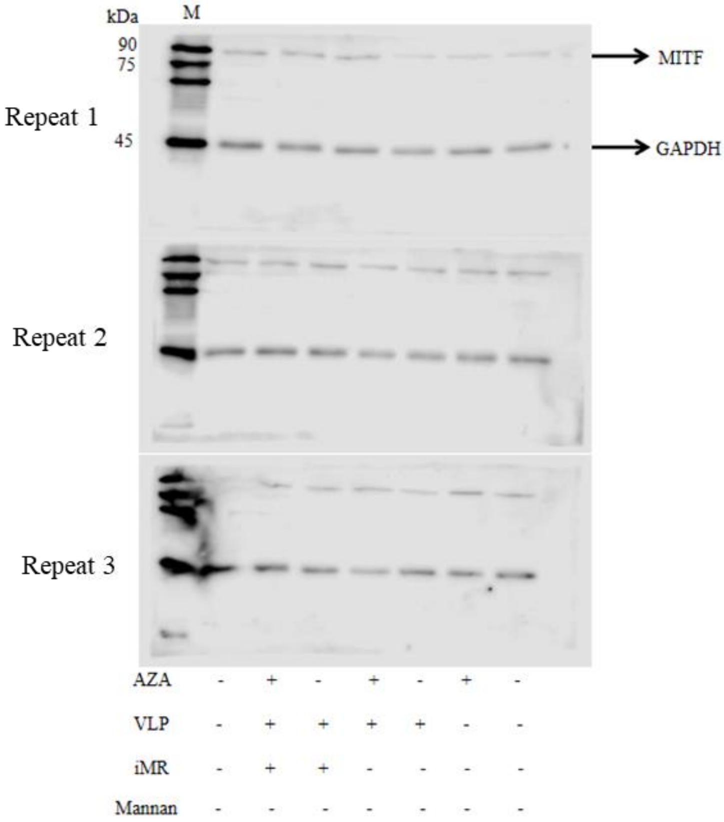

MITF

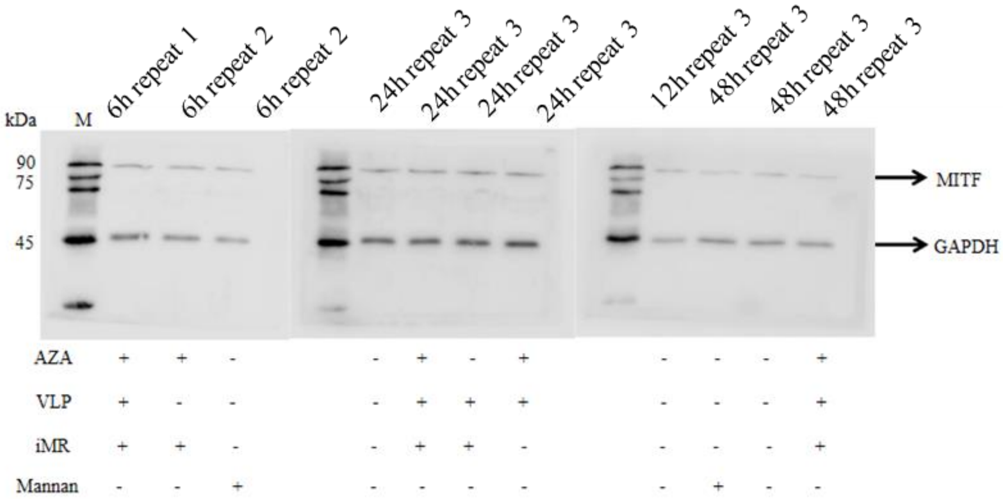

NF-κB、GAPDH 6h :

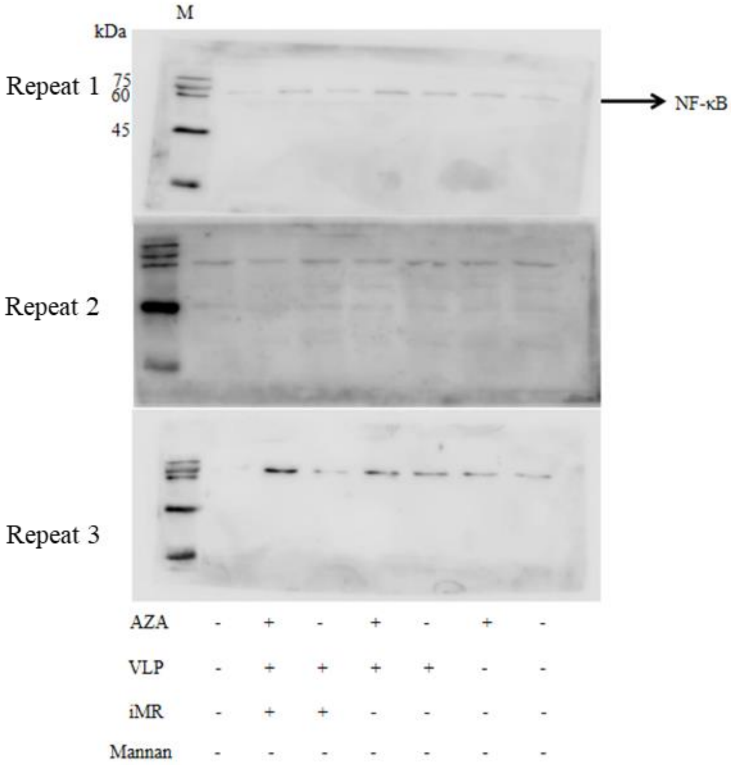

NF-κB、GAPDH　12h :

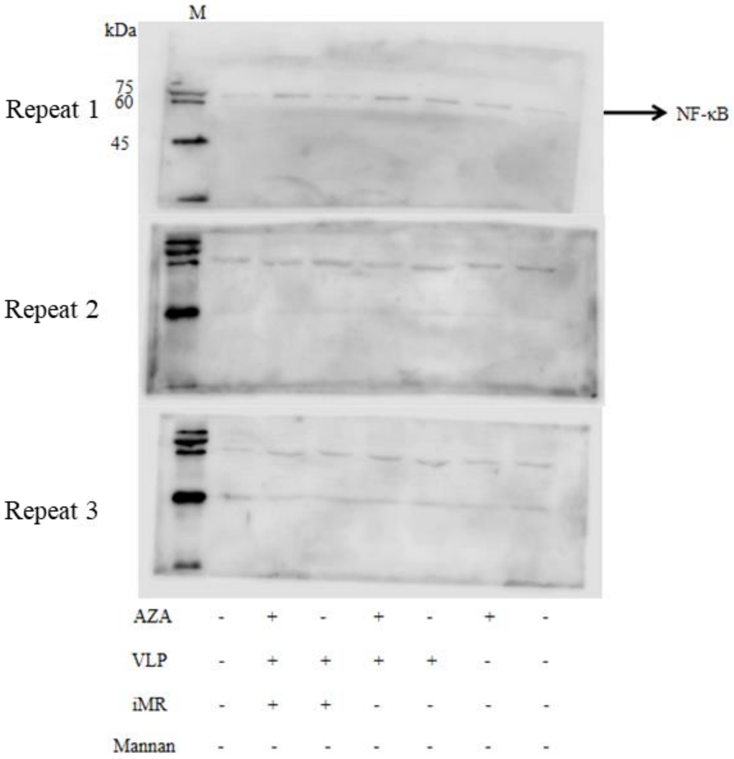

NF-κB、GAPDH　24h :

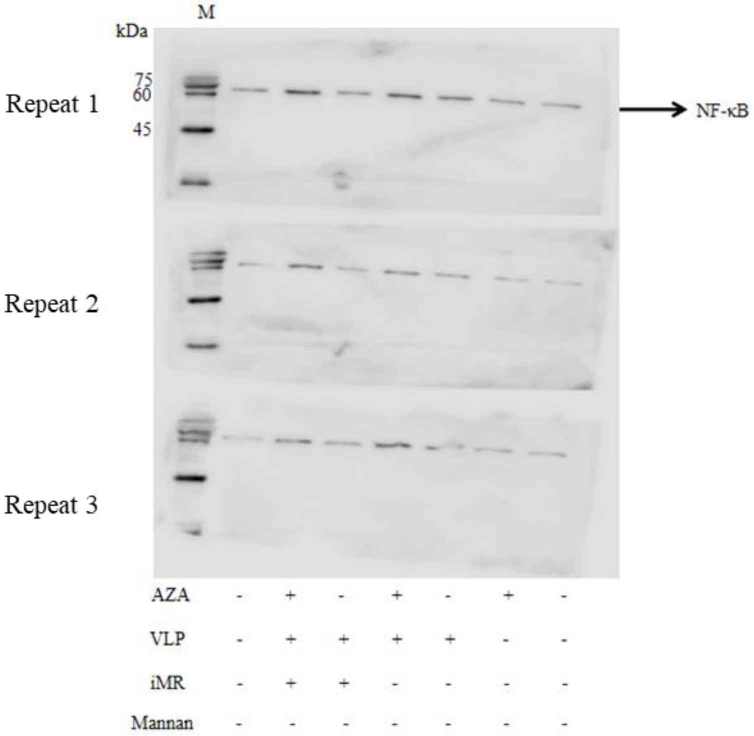

NF-κB、GAPDH　48h :

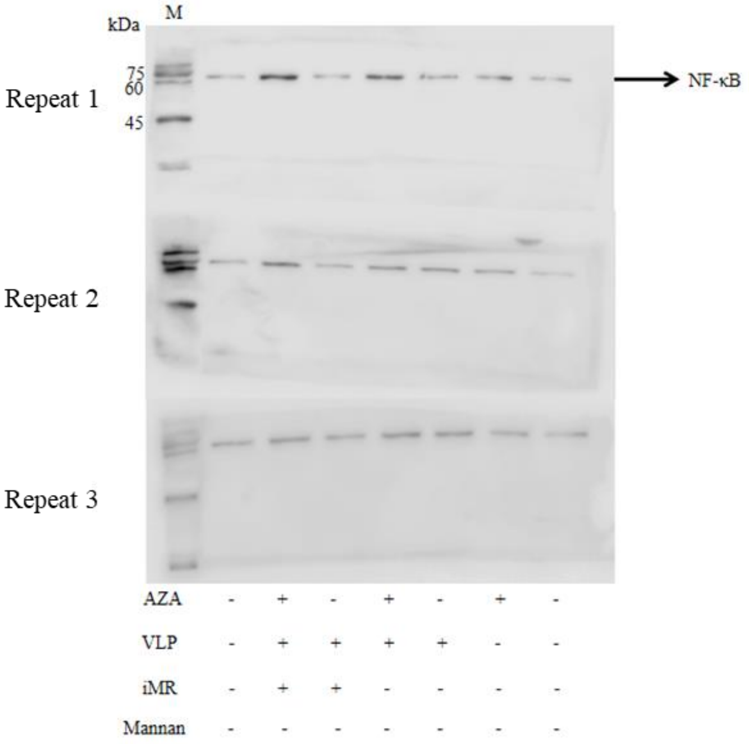

M
kDa
75
60
45
Repeat 1
Repeat 2
Repeat 3
NF-κB
AZA - + - + - + -
VLP - + + + + - -
iMR - + + - - - -
Mannan - - - - - - -

NF-κB:

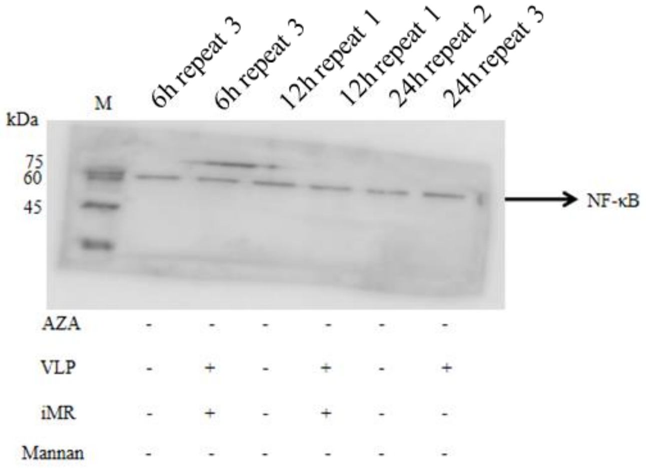

6h repeat 3
6h repeat 3
12h repeat 1
12h repeat 1
24h repeat 2
24h repeat 3
M
kDa
75
60
45
NF-κB
AZA - - - - - -
VLP - + - + - +
iMR - + - + - -
Mannan - - - - - -

GATA-2、GAPDH 6h :

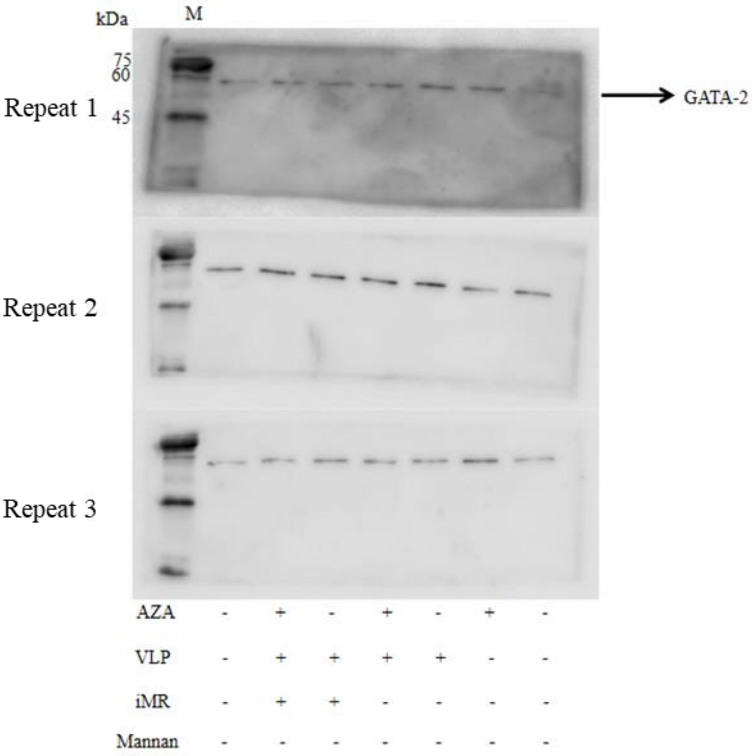

GATA-2、GAPDH 12h :

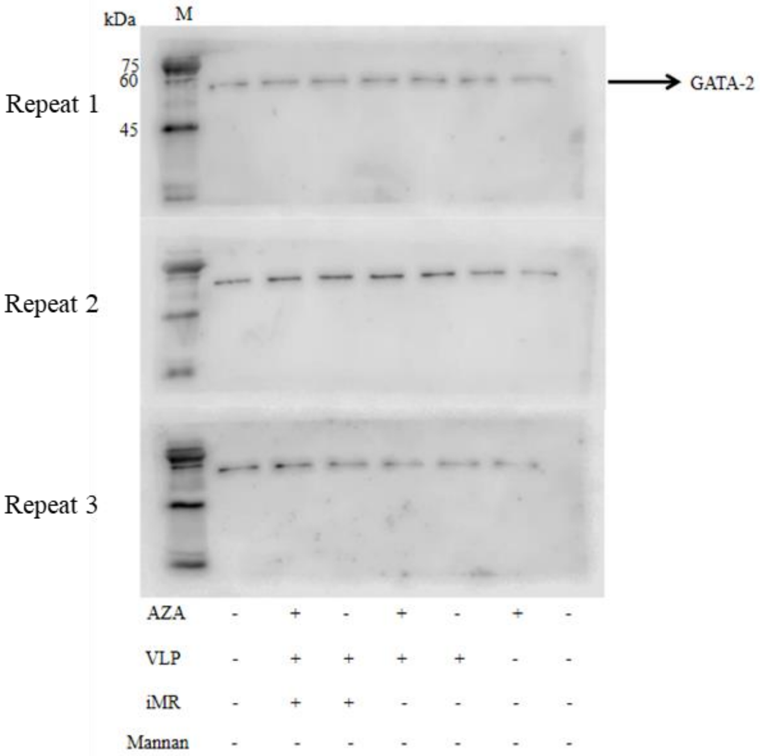

GATA-2、GAPDH　24 h :

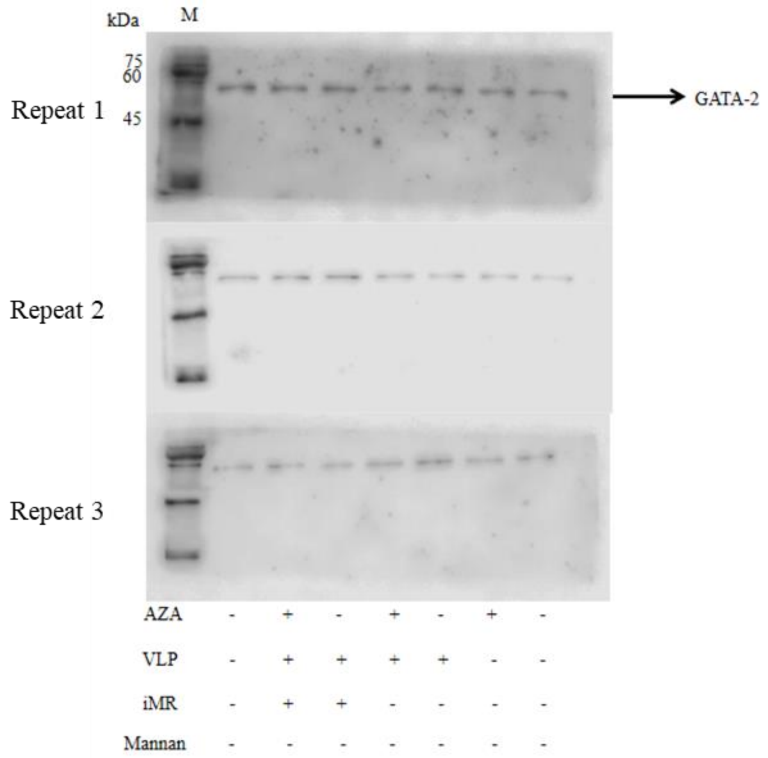

GATA-2、GAPDH　48 h :

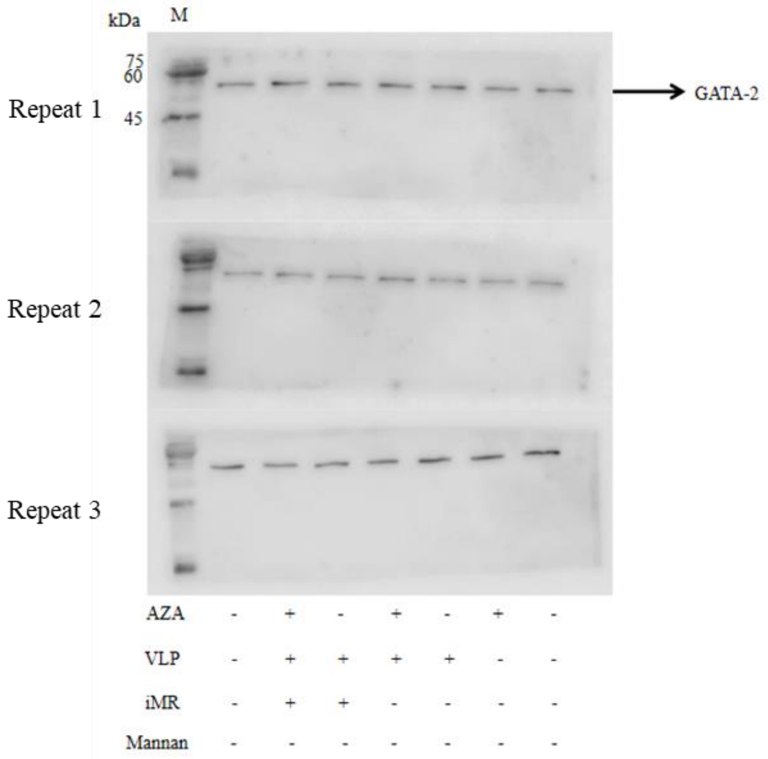

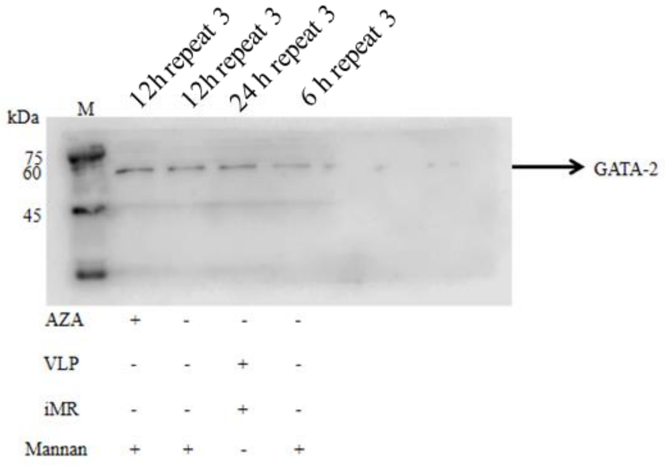

Figure S1B Determination of transcription factors expression by western blot. Data were representative of 3 experiments. “Repeat” represents one of the 3 experiments. Three PVDF membranes were put into the chemiluminescence imaging system for exposure at the same time.
