# Supplementary material for: DNA Methylation Negatively Regulates Gene Expression of Key Cytokines Secreted by BMMCs Recognizing FMDV-VLPs
Source: Int J Mol Sci. 2024 Oct 9;25(19):10849. doi: 10.3390/ijms251910849 (PMC11477203; doi:10.3390/ijms251910849)
Supplement: Supplementary file 1 [file ijms-25-10849-s001.zip › Figure S2.pdf]

A: GATA-2

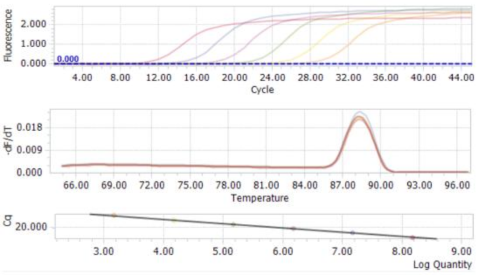

R^2=1.00 Efficiency=1.92 Slope=-3.5280

B: IL-10

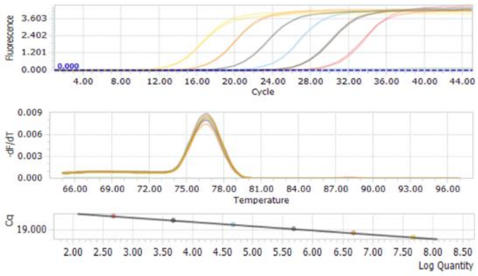

R^2=1.00 Efficiency=1.99 Slope=-3.3421

C: IL-13

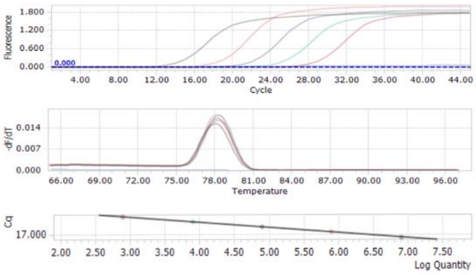

R^2=1.00 Efficiency=1.91 Slope=-3.5510

D:MITF

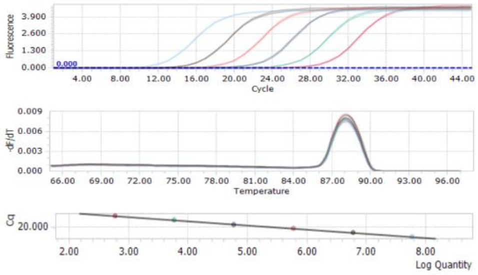

R^2=1.00 Efficiency=1.97 Slope=-3.3944

E:TNF-α

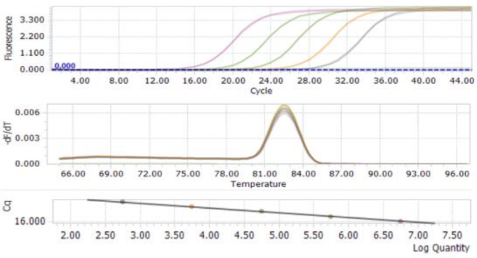

R^2=1.00 Efficiency=2.01 Slope=-3.3015

F:IL-6

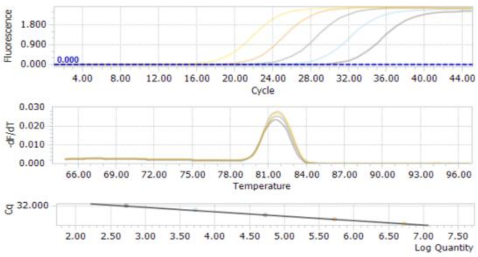

R^2=1.00 Efficiency=1.93 Slope=-3.5050

G:NF-κB

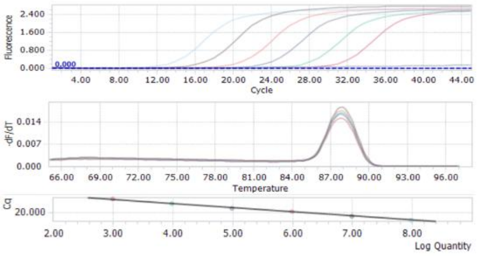

R^2=1.00 Efficiency=1.90 Slope=-3.5763

H:GADPH

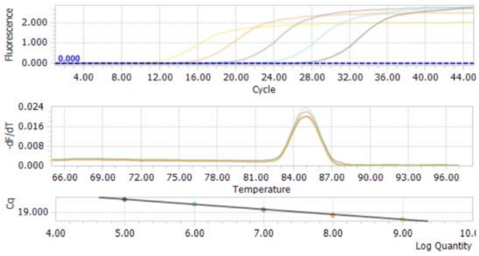

R^2=1.00 Efficiency=1.73 Slope=-4.2015

Supplementary Figure-S2. Standard curves of real-time quantitative PCR
