# Supplementary material for: DNA Methylation Negatively Regulates Gene Expression of Key Cytokines Secreted by BMMCs Recognizing FMDV-VLPs
Source: Int J Mol Sci. 2024 Oct 9;25(19):10849. doi: 10.3390/ijms251910849 (PMC11477203; doi:10.3390/ijms251910849)
Supplement: Supplementary file 1 [file ijms-25-10849-s001.zip › Figure S3.pdf]

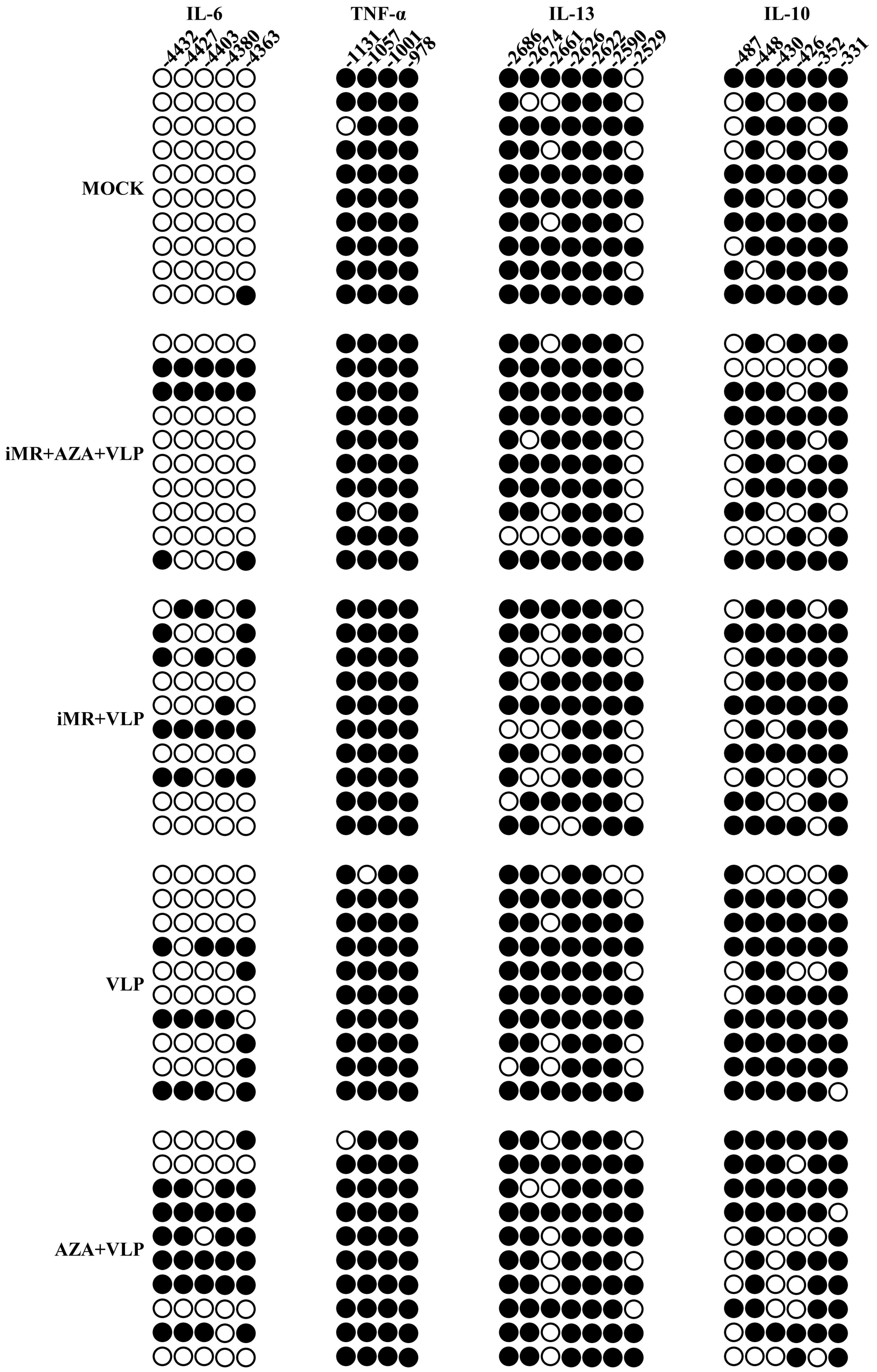

Supplementary Figure-S3. Methylation pattern of CpG sites in the amplified region of cytokines promoter. The circles indicated methylation status. Black solid circles (●) indicated methylated status, and black hollow circles (○) indicated unmethylated status. The top numbers indicated the sites relative to the 5'-UTR.
