# Supplementary material for: DNA Methylation Negatively Regulates Gene Expression of Key Cytokines Secreted by BMMCs Recognizing FMDV-VLPs
Source: Int J Mol Sci. 2024 Oct 9;25(19):10849. doi: 10.3390/ijms251910849 (PMC11477203; doi:10.3390/ijms251910849)
Supplement: Supplementary file 1 [file ijms-25-10849-s001.zip › Figure S4.pdf]

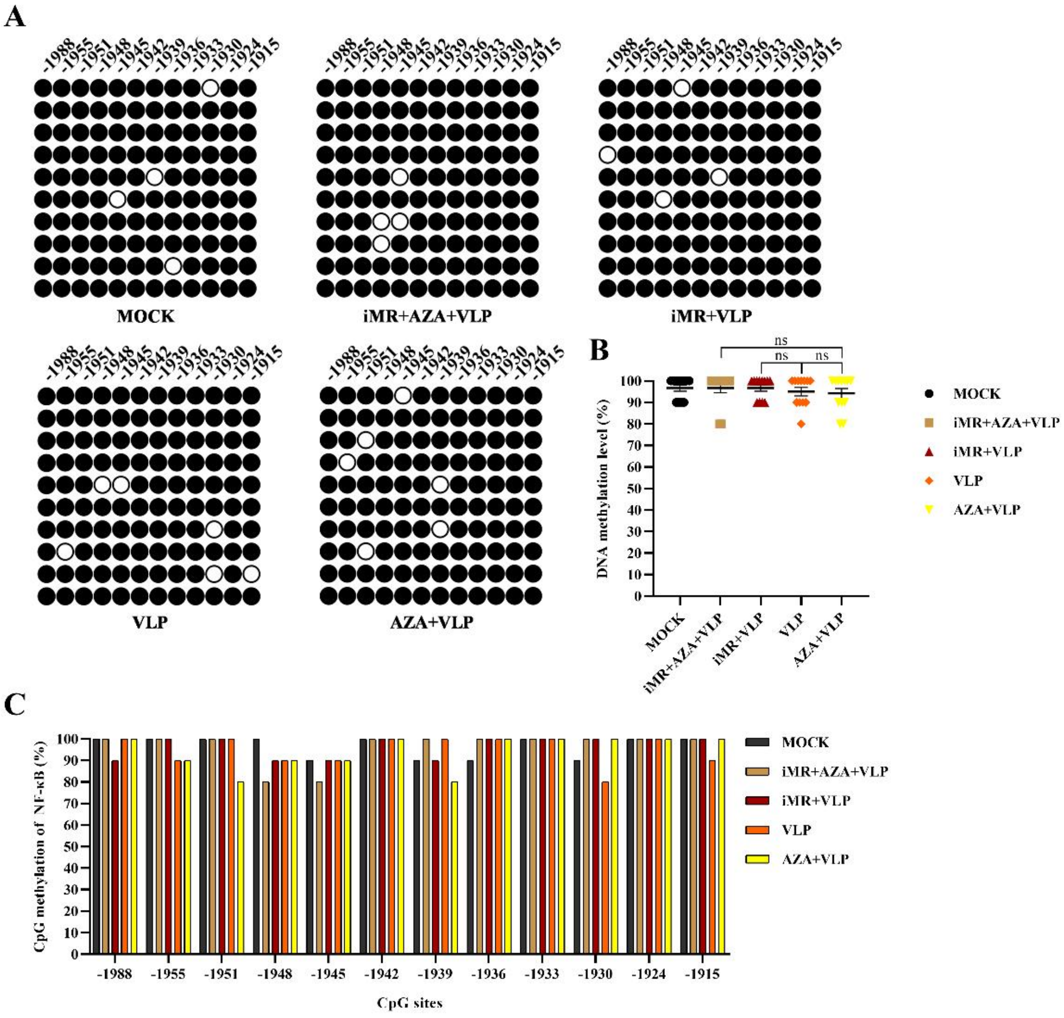

Supplementary Figure-S4. DNA methylation analysis of NF-κB promotor. (A) Methylation status of each CpG site in the amplified region of NF-κB promoter. The circles indicated methylation status. Black solid circles (●) indicated methylated status, and black hollow circles (○) indicated unmethylated status. The top numbers indicated the sites relative to the 5'-UTR. (B) Methylation rate of different groups. Each data represents the average of DNA methylation rate in each locus. DNA methylation rate in each locus=number of methylations at that locus / number of sequences measured. One-way ANOVA was carried out. ns indicates $P>0.05$. (C) Methylation rate of different sites in the promoter region of NF-κB. The abscissa number indicates the location of each CpG locus relative to the 5'-UTR. DNA methylation rate in each locus=number of methylations at that locus / number of sequences measured.
